# Effects of a dynamically changing response set overlap on *n* − 2 repetition costs

**DOI:** 10.1007/s00426-023-01816-w

**Published:** 2023-03-22

**Authors:** Juliane Scheil, Thomas Kleinsorge

**Affiliations:** Leibniz Research Centre for Working Environment and Human Factors, Ardeystraße 67, 44139 Dortmund, Germany

## Abstract

*N* − 2 repetition costs are a marker for inhibition processes during task switching that are supposed to reduce interference from currently irrelevant information. The present study aimed at elucidating effects of response set overlap on *n* − 2 repetition costs while keeping stimulus set overlap constant. For this purpose, each task was associated with two different response sets. The relevant response set was visually cued in every trial. *N* − 2 repetition costs were present when the response set overlapped from trial *n* − 2 to trial *n* − 1. In contrast, they were abolished when the response set switched. This result is interpreted in terms of stronger interference for overlapping response sets that need to be inhibited to a high degree, resulting in large *n* − 2 repetition costs. Furthermore, the present results support the notion that two means for interference reduction, task inhibition and task shielding, are deployed in a flexible way depending on environmental demands.

**Supplementary Information:**

The online version contains supplementary material available at 10.1007/s00426-023-01816-w.

## Introduction

In our daily lives, we constantly adapt our behavior in accordance with environmental changes. This flexible control of behavior is one of the core functions of human cognition, and the task switching paradigm is suited to investigate it under lab conditions (see Kiesel et al., 2010; Vandierendonck et al., 2010, for reviews). When switching among different tasks, we are confronted with the need to activate relevant information on the one hand and, at the same time, prevent interference from currently irrelevant information. While the former may be achieved by activating some kind of task set, the latter may be accomplished by inhibitory processes (Koch et al., 2010).

One of the most straightforward indications for the involvement of inhibition in task switching is *n* − 2 repetition costs (Mayr & Keele, 2000). When switching among at least three different tasks A, B, and C, reactions are slower and more error-prone when the task in the current trial equals the task in trial *n* − 2 (sequence of type ABA) compared to two consecutive switches to another task (sequence CBA). This effect is typically explained by the occurrence of task inhibition after each switch trial that persists for some time and, therefore, has to be overcome when the current task was inhibited after trial *n* − 2.

During the last decades, *n* − 2 repetition costs have been investigated using different paradigms using different kinds of tasks (see Koch et al., 2018, for a recent review). It has been shown that their size and presence vary depending on characteristics of the experimental environment. On the one hand, this holds for factors that affect all tasks and trials to the same degree, for example, which type of cues are used to identify the currently relevant task (abstract, verbal, or direct cues, Gade & Koch, 2014). On the other hand, *n* − 2 repetition costs are affected by factors that exert their influence on the level of individual tasks, like task dominance (Jost et al., 2017), or on the level of features of single trials, like the presentation of different types of distractors (Kuhns et al., 2007). Furthermore, the same factor may affect *n* − 2 repetition costs differentially, depending on the level on which it is manipulated. For example, the introduction of no-go trials diminishes *n* − 2 repetition costs if no-gos are present for all tasks (Schuch & Koch, 2003), but enhances *n* − 2 repetition costs if they are tied to only one of the tasks (Scheil & Kleinsorge, 2022). Another example is the occurrence of task repetitions that has been shown to affect *n* − 2 repetition costs in different ways depending on whether all tasks repeat to the same degree (Philipp & Koch, 2006) whether the proportion of repetitions varies for the three tasks (Scheil & Kleinsorge, 2019). Taken together, the picture that emerges shows inhibition processes during task switching as being not rigid and invariant across trials and tasks. Rather, it seems that inhibition is exerted in a flexible way, depending on characteristics of the experimental environment that affect the need for inhibition to reduce interference.

One particular factor that has been shown to affect inhibition during task switching is the degree of overlap between tasks or task sets. Broadly, it can be assumed that highly overlapping tasks cause greater interference due to crosstalk that hampers performance. As a consequence, inhibition is levelled up, causing higher *n* − 2 repetition costs. For example, Arbuthnott (2005) showed that *n* − 2 repetition costs are abolished when the stimuli for different tasks are uniquely located in space. This was interpreted in terms of higher discriminability that reduced the need for inhibition. Further evidence comes from Gade and Koch (2007) who introduced a fourth task with unique stimuli. The degree of response set overlap was varied across experiments. *N* − 2 repetition costs were abolished when responses did not overlap but were present with overlapping response sets. The authors concluded that overlapping response sets cause competition among tasks and, therefore, trigger inhibition. However, as the fourth task was always associated with unique stimuli, it is possible that not only the degree of overlap among responses but also among stimuli had an influence on *n* − 2 repetition costs. In the present study, we aimed at further investigating effects of response set overlap while using the same stimuli for all tasks. For doing this, we used a standard procedure with three tasks that never repeated across subsequent trials. Each task was associated with two different response sets. Which set was the relevant one in a certain trial was indicated by the color of the task cue. Using this procedure, we were able to manipulate the overlap of response sets on a trial-by-trial basis. Furthermore, this provided us with a baseline measure equivalent to the “standard” paradigm, in which the same response set was used throughout an entire three-trial sequence. Following previous research, we hypothesized that *n* − 2 repetition costs will be larger for overlapping than for distinct response sets. Please note that the term “distinct” refers to the mere spatial position of the two response sets, but not to their representation within the task set. In accordance with the previous literature, we assume a dissociation between the anatomical position of a response and its abstract coding. Previous literature suggests that there is a differentiation between these two kinds of representations. For example, Nicoletti et al. (1984) let participants cross their hands when responding to spatial stimuli. They could show that the relative correspondence of stimulus and response (i.e., the location of the stimulus and of the responding hand) affected task performance, while the absolute correspondence (i.e., the hemisphere of the body the responding hand belongs to) did not (see also Heister et al., 1990, for a review). We assume that in the present study, the two response sets are distinctly represented with respect to their anatomical position. However, with regard to their relative correspondence, we assume that both response sets are represented together within the task set. For example, the left response key of both response sets is assumed to be coded as “left”, irrespective of the responding effector.

Previous research from our lab has shown that fluctuations in the presence and size of *n* − 2 repetition costs may be accompanied by different amounts of task shielding (Scheil & Kleinsorge, 2020). Like inhibition, task shielding is assumed to be a mechanism for reducing interference during task switching (e.g., Dreisbach, 2012). On a methodological level, task shielding can be measured by assessing effects of stimulus congruency (Schuch & Grange, 2019). When the same stimuli are used for different tasks, a stimulus may afford the same response for all tasks (congruent stimuli). Alternatively, it may be associated with different responses, depending on task identity (incongruent stimuli). Based on previous research, we assume that task shielding is effective especially when tasks or task sets are easily discriminable, that is, when they are associated with different response sets.

## Method

### Participants

32 subjects (5 male) with a mean age of 24.0 years (range: 19–32) participated. All had normal or corrected-to-normal vision. For power estimation, we used the effect size of Experiment II of Scheil (2016), where the effect of changes on the response level on *n* − 2 repetition costs was investigated. Using G-Power (Faul et al., 2007), this yielded a power estimation of 0.99 for this sample.

### Stimuli, tasks, and apparatus

Stimuli consisted of two different shapes (× and +) presented in solid or dashed line and with a size of either 3 × 3 cm or 6 × 6 cm. Task cues consisted of a diamond, square, or triangle surrounding the position of the imperative stimulus with a size of about 7 × 7 cm. Participants switched among three perceptual decision tasks in which they had to judge the stimuli regarding their size (large vs. small, indicated by the diamond), line type (solid or dashed, indicated by the square), or their shape (× or + , indicated by the triangle). A trial illustration can be found in the online supplemental material. All tasks occurred with equal frequency. Immediate stimulus repetitions were not allowed. Stimuli were presented centrally on a light-grey background. Two sets of response keys were used throughout the experiment. Participants had to press the “*y*”-key of a German QWERTZ keyboard with the left middle finger and the “*x*”-key with the left index finger. The “.”-key had to be pressed with the right index finger and the “–”-key with the right middle finger. Which response set had to be used was indicated by the color of the task cue. For half of the participants, a yellow task cue indicated the left response set, whereas a blue task cue indicated the right set. For the other half of participants, the meaning of the colors was reversed. The response set as well as the set transition (repetition or switch) was pseudo-randomized across tasks and task sequences. Participants had to press the left key of either response set for small, dashed, and x-shaped stimuli and the right key for large, solid, and + -shaped stimuli. Only stimuli were used that were either fully congruent (i.e., affording the same relative response position for all three tasks, defined as a left or right keypress irrespective of the response set) or fully incongruent (i.e., affording a different relative response position for the currently relevant task than for the two currently irrelevant tasks). Viewing distance was not controlled but approximated 60 cm.

### Design and procedure

After giving informed consent, participants were provided with on-screen instructions in which the tasks and the meaning of the task cues were explained. Instructions emphasized speed as well as accuracy. The experiment was run in a single session that took about 70 min. It started with a practice block of 72 trials. In this block, all tasks were practiced separately for 14 trials each. After that, 30 mixed trials without task repetitions followed. The test session consisted of 18 experimental blocks of 72 trials each.

Each trial began with the presentation of a fixation mark for 300 ms, followed by the task cue which was presented for 600 ms. After that, the cue disappeared and the imperative stimulus was presented for 2500 ms or until the participant’s response. In case of an error, error feedback was presented for additional 1000 ms; in case of RTs slower than the RT deadline of 2500 ms, RT feedback was presented for additional 1000 ms.

## Results

### Main analysis

The practice block as well as the first three trials of each block were not analyzed. Furthermore, sequences involving an error in trials *n* − 2 or *n* − 1 were excluded. From RT analyses, errors in the current trial were also excluded. As dependent measure, linear integrated speed-accuracy scores (LISAS, Vandierendonck, 2018) were calculated and analyzed using a repeated-measures ANOVA with the within-subjects factors Task Sequence (CBA vs. ABA), Set-Switch (repetition vs. switch of the response set from trial *n* − 1 to trial *n*), lag_Set-Switch (repetition vs. switch of the response set from trial *n* − 2 to trial *n* − 1), and Congruency in trial *n*^1^ (congruent vs. incongruent, defined in terms of relative response positions).

The main effect of Task Sequence was significant, *F* (1, 31) = 18.00, *p* < 0.001, η_p_^2^ = 0.37, because LISAS were higher for ABA sequences (948) than for CBA sequences (921). Furthermore, the main effect of Set-Switch was significant, *F* (1, 31) = 125.82, *p* < 0.001, η_p_^2^ = 0.80, due to higher LISAS for set switches (1003) compared to set repetitions (866). The main effect of lag_Set-Switch was significant as well, *F* (1, 31) = 30.44, *p* < 0.001, η_p_^2^ = 0.50, because switches from trial *n* − 2 to trial *n* − 1 were associated with higher LISAS (957) than repetitions (912). The main effect of Congruency was significant, *F* (1, 31) = 17.62, *p* < 0.001, η_p_^2^ = 0.36, because incongruent stimuli led to higher LISAS (957) than congruent ones (912). This effect was modulated by Set-Switch, *F* (1, 31) = 6.54, *p* < 0.05, η_p_^2^ = 0.17, because the congruency effect was larger for set switches (mean difference of 58) than for set repetitions (31). Set-Switch and Task Sequence interacted, *F* (1, 31) = 6.88, *p* < 0.05, η_p_^2^ = 0.18. Significant *n* − 2 repetition costs occurred when the same task set had to be performed in trials *n* − 1 and *n* (47, *p* < 0.001, Tukey corrected), while no costs were visible after set switches (7, *p* = 0.91). Task Sequence was furthermore modulated by lag_Set-Switch, *F* (1, 31) = 21.93, *p* < 0.001, η_p_^2^ = 0.41: significant *n* − 2 repetition costs were visible if the same response set had to be used in trials *n* − 2 and *n* − 1 (50, *p* < 0.001), while no costs occurred after set switches (4, *p* = 0.97). Task Sequence and Congruency interacted, *F* (1, 31) = 5.10, *p* < 0.05, η_p_^2^ = 0.14. Significant *n* − 2 repetition costs could be observed for incongruent stimuli (43, *p* < 0.01), while they were not significant if the stimulus was congruent (11, *p* = 0.70).

**Fig. 1:**
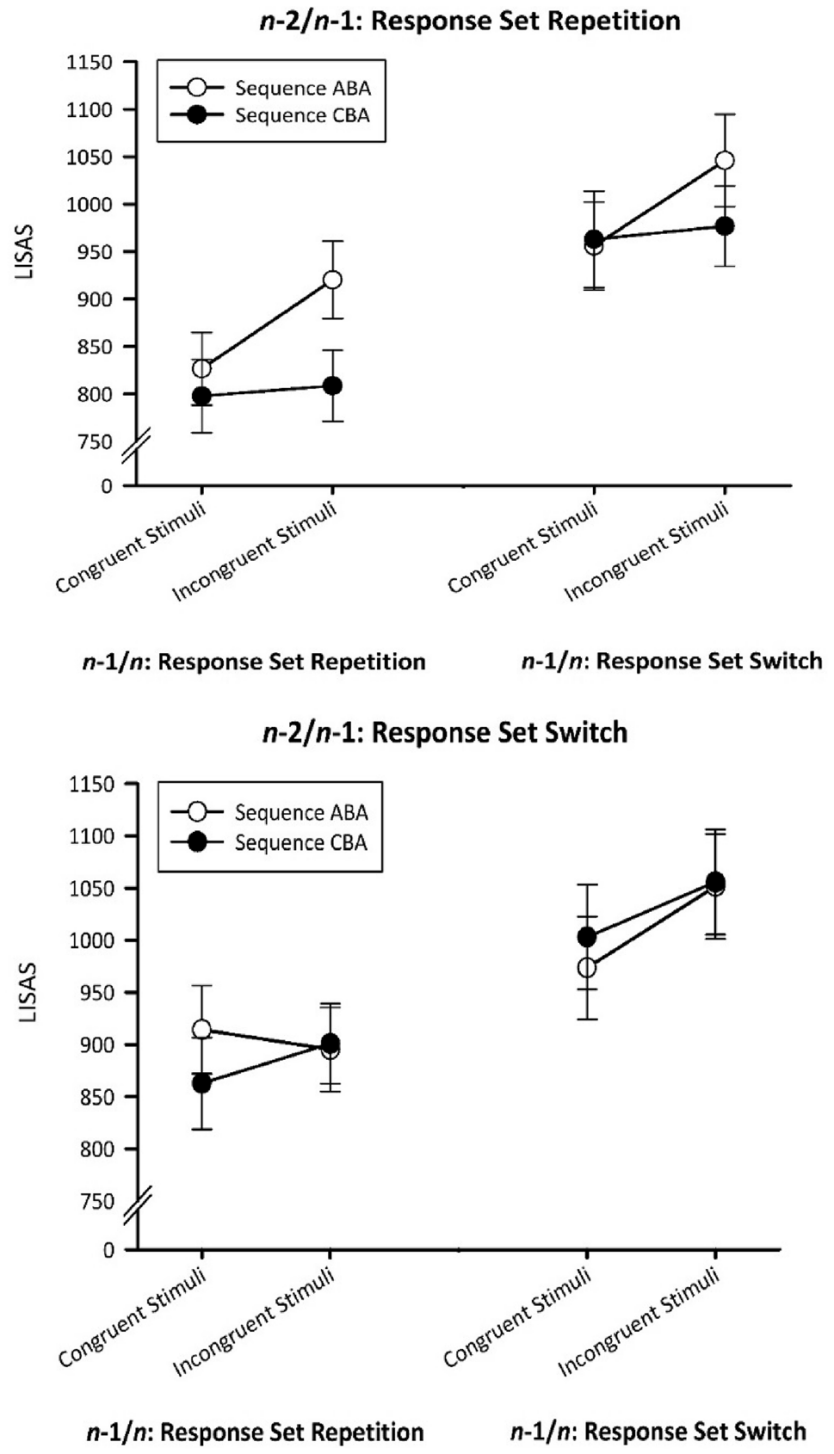
Mean LISAS as a function of Set-Switch, lag_Set-Switch, congruency, and task sequence. Error bars represent SEM

Coming to the three-way interactions, Task Sequence was modulated by lag_Set-Switch and Congruency, *F* (1, 31) = 17.46, *p* < 0.001, η_p_^2^ = 0.36. For response set switches from trial *n* − 2 to trial *n* − 1, no *n* − 2 repetition costs were visible, irrespective of stimulus congruency (21, *p* = 0.98 for congruent and -5, *p* = 0.99 for incongruent stimuli, respectively). After response set repetitions, significant *n* − 2 repetition costs were visible for incongruent stimuli (90, *p* < 0.001), whereas no cost occurred for congruent ones (11, *p* = 0.98). Furthermore, the interaction of Set-Switch, lag_Set-Switch, and Congruency was significant, *F* (1, 31) = 5.40, *p* < 0.05, η_p_^2^ = 0.15. For response set switches from trial *n* − 1 to trial *n*, a significant congruency effect was visible irrespective of lag_Set-Switch (52, *p* < 0.01 for set repetitions from trial *n* − 2 to *n* − 1 and 66, *p* < 0.001, for switches, respectively). For response set repetitions that were preceded by another set repetition, the congruency effect was significant as well (52, *p* < 0.01), but it vanished for set repetitions that were preceded by a set switch (10, *p* = 0.99). This was mainly driven by fast ABA sequences in this condition, which can be derived from the significant four-way interaction, *F* (1, 31) = 4.41, *p* < 0.05, η_p_^2^ = 0.12 (see Fig. 1)^2^. When the response set repeated from trial *n* − 2 to trial *n* − 1, the data pattern equaled the three-way interaction, showing significant *n* − 2 repetition costs for incongruent stimuli (*p* < 0.001 for response set repetitions from *n* − 1 to *n* and *p* < 0.01 for response set switches), while no costs were visible if the stimulus was congruent (*p* = 0.85 and *p* = 0.99, respectively). When the response set switched from trial *n* − 2 to trial *n* − 1 but repeated from trial *n* − 1 to trial *n*, there was a trend for the occurrence of *n* − 2 repetition costs for congruent stimuli (*p* = 0.09), while they were not present for incongruent stimuli (*p* = 0.99). For two successive response set switches, no *n* − 2 repetition costs were visible irrespective of Congruency (both *p*’s > 0.82).

### Supplemental analyses

One potential confound that has to be mentioned related to the identity of the imperative stimulus. Specifically, there is only one congruent stimulus for left and right responses, respectively, because only two stimuli are congruent with respect to all three tasks (the small dashed × for the left response key and the large solid + for the right response key). In contrast, there were six incongruent stimuli, resulting from two incongruent stimuli (one for left and right response key, respectively) for each task. As a consequence, based on the main analysis alone, one cannot exclude that stimulus identity has an impact on the data pattern. In the same vein, episodic retrieval of stimulus features may have affected the results. This factor has been shown to influence *n* − 2 repetition costs due to an inflation of costs when the stimulus repeats from trial *n* − 2 to trial *n* in ABA sequences (Grange et al., 2017). To check whether the present data pattern was really driven by stimulus congruency instead of episodic retrieval, we subjected mean individual LISAS to a repeated-measures ANOVA with the within-subjects factors Task Sequence (CBA vs. ABA), Set-Switch (repetition vs. switch of the response set from trial *n* − 1 to trial *n*), lag_Set-Switch (repetition vs. switch of the response set from trial *n* − 2 to trial *n* − 1), and Stimulus Repetition from trial *n* − 2 to trial *n* (repetition vs. switch). There was main effect of Stimulus Repetition, *F* (1, 31) = 5.77, *p* < 0.05, η_p_^2^ = 0.16, because LISAS were higher for stimulus switches (940) than for stimulus repetitions (916). All other effects involving this factor were not significant, including the interaction with Task Sequence (all *p*’s > 0.15). Although the present study was not designed for investigating the effects of episodic retrieval, this does not support the notion that the present data pattern was affected by stimulus features or episodic retrieval. Instead, we conclude that the results were driven by effects of stimulus congruency.

In a second step, we checked whether response repetition effects had an influence on the present data pattern. It is known from previous research that response repetitions have impacts on different effects in task switching, essentially on switch costs (cf., e.g., Kleinsorge, 1999; Rogers & Monsell, 1995). As no task repetitions were included in the present design, this interaction is of no significance here. Nevertheless, it is possible that this factor affects the data pattern in other ways. However, adding the response repetition factor to the main ANOVA decreased the mean number of observations per cell (down to 18 in some conditions). Therefore, two ANOVAs were run, each leaving one factor out. Only effects involving the response repetition factor are reported.

First, mean individual LISAS were subjected to a repeated-measures ANOVA with the within-subjects factors Congruency in trial *n* (congruent vs. incongruent), Set-Switch (repetition vs. switch of the response set from trial *n* − 1 to trial *n*), lag_Set-Switch (repetition vs. switch of the response set from trial *n* − 2 to trial *n* − 1), and Response Transition from trial *n* − 1 to trial *n* (repetition vs. switch). The main effect of Response Transition was marginally significant, *F* (1, 31) = 4.14, *p* = 0.0504, because LISAS tended to be higher for response switches (946) than for repetitions (931). Response Transition interacted with Set-Switch, *F* (1, 31) = 12.38, *p* < 0.01, η_p_^2^ = 0.29, because the response repetition effect was present for response set repetitions (mean difference in LISAS of 38) but vanished when the response set switched (mean difference of −6). Congruency and Response Transition interacted, *F* (1, 31) = 8.42, *p* < 0.01, η_p_^2^ = 0.21, because congruency effects were larger for response repetitions (mean difference in LISAS of 57) but decreased for response switches (mean difference of 24). All other factors involving response transition were not significant, all *p*’s > 0.13.

Second, mean individual LISAS were subjected to a repeated-measures ANOVA with the within-subjects factors Task Sequence (CBA vs. ABA), Set-Switch (repetition vs. switch of the response set from trial *n* − 1 to trial *n*), lag_Set-Switch (repetition vs. switch of the response set from trial *n* − 2 to trial *n* − 1), and Response Transition from trial *n* − 1 to trial *n* (repetition vs. switch). There was a main effect of Response Transition, *F* (1, 31) = 4.84, *p* < 0.01, η_p_^2^ = 0.14, because LISAS were higher for response switches (949) than for repetitions (934). Response Transition and Set-Switch interacted, *F* (1, 31) = 16.12, *p* < 0.001, η_p_^2^ = 0.24. As it was the case in the ANOVA including the Congruency factor, a response repetition effect was present for response set repetitions (mean difference in LISAS of 41) but vanished when the response set switched (mean difference of −10). All other factors including Response Transition were not significant, all *p*’s > 0.10. Taken together, we conclude that response repetition effect does not modulate the present results of interest, that is, effects of response set overlap.

## Discussion

The present study aimed at further investigating effect of response set overlap on *n* − 2 repetition costs in task switching. For this purpose, each task was associated with two response sets, with the relevant set being indicated by the task cue.

The main finding of the present study is the significant modulation of *n* − 2 repetition costs by response set transition: significant *n* − 2 repetition costs were visible after a response set repetition, while no costs occurred after a response set switch. This result is in line with Gade and Koch (2007) who could show that *n* − 2 repetition costs decrease when task set structure does not overlap. However, Gade and Koch implemented a fourth task with a unique stimulus set and varying degrees of response set overlap with respect to the other three tasks that always shared the same response set. As a consequence, it is not possible to solely attribute the effects to the response set, because the fourth task differed from the other three even in the baseline condition. The present study fills this gap. As all three tasks overlapped with regard to the stimulus set, effects can clearly be ascribed to the overlap of response set.

In line with Gade and Koch (2007), the reduction of *n* − 2 repetition costs for non-overlapping response sets can be interpreted in terms of reduced interference when the response set switches. As the relevant response set was indicated by the task cue, participants knew from an early task preparation stage on that the response set of the previous trial will not be relevant in the current trial. As a consequence, no interference from the previous response set may hamper performance, so there is no need to inhibit this (now irrelevant) response set. This leads to a reduced amount of inhibition being released, and, consequently, to reduced *n* − 2 repetition costs. Of course, this does not mean that there is no inhibition involved at all, because there are still other parts of the task set that may cause interference and have to be inhibited. However, as zero *n* − 2 repetition costs can be assumed to be due to a reduced amount of inhibition and not to no inhibition at all (see, e.g., Grange et al., 2013), the present results are well in line with a reduced amount of inhibition after response set switches. This in turn means that the cognitive system is not only able to flexibly adjust the amount of inhibition that is triggered in every trial, but also to selectively inhibit certain parts of the task set instead of the task set as a whole. This assumption is in line with Houghton et al. (2009) who proposed that inhibition targets the aspect of the task set that bears the highest conflict potential.

Furthermore, the present results indicate an interaction of task sequence and stimulus congruency in trial *n*. LISAS were especially high in ABA sequences with incongruent stimuli. In this condition, the relevant task set is still in an inhibited state when the cognitive system is confronted with an incongruent stimulus that bears a high risk of task confusion. Therefore, it makes sense that task performance is especially impaired in this condition.

The interaction of task sequence and stimulus congruency replicates the previous findings of Scheil and Kleinsorge (2020) who also observed *n* − 2 repetition costs to be dependent on stimulus congruency in conditions in which the tasks did not repeat, as it was the case in the present study. As soon as task repetitions were present, the interaction vanished. This was interpreted in terms of task inhibition and task shielding being active in a complementary way to reduce interference from currently irrelevant tasks: When a task repeats, it is easily shielded against distracting information due to a higher activation of the relevant task set. As a consequence, irrelevant stimulus features have only a minor effect on task performance, so congruency effects diminish. For task switches, on the other hand, task shielding has to be relaxed, making the task set more vulnerable for irrelevant information and causing larger congruency effects (e.g., Dreisbach & Wenke, 2011). Thus, task shielding is especially useful for interference reduction in the case of a task repetition. For inhibition, a different pattern emerges. As inhibition in task switching can be assumed to be triggered by the preparation for a different task (Scheil & Kleinsorge, 2014), it is especially useful for interference reduction in the case of a task switch. As a consequence, *n* − 2 repetition costs are visible for task switches but reduced when task repetitions are allowed (Philipp & Koch, 2006; Scheil & Kleinsorge, 2019, 2020). Thus, it seems that interference reduction in task switching is brought about by either task inhibition or task shielding, depending on which process corresponds better with the characteristics of the experimental situation. This can be taken as an example of how flexible our cognitive system reacts to changing environmental demands.

Coming back to the present results, the flexible interplay of task inhibition and task shielding was furthermore visible in effects of switching response sets. *N* − 2 repetition costs were present when the response set repeated but were absent when the response set switched. During task preparation, information of the previously relevant task set is inhibited. However, if the response set switches, there is no need to inhibit response-related information of the previously relevant task set, because this information does not interfere with the task set that is relevant in the current trial. As a consequence, the release of inhibition is reduced, minimizing *n* − 2 repetition costs. If the response set repeats, on the other hand, this leads to a full-blown inhibition process that causes high *n* − 2 repetition costs. In contrast, the influence of response set transition on congruency effects showed a reversed picture: large congruency effects were visible for response set switches that were reduced when the response set repeated. Again, it can be argued that repetitions of at least parts of the task set aids task shielding, which in turn reduces congruency effects. This furthermore supports the notion of task inhibition and task shielding being active in a flexible way to reduce interference of currently irrelevant information.

An additional note has to be made regarding the causal relationship among the factors. More precisely, this relates to the question whether response set overlap and congruency have an effect on *n* − 2 repetition costs, or whether response set overlap and the task sequence have an effect on congruency. It is important to point out that *n* − 2 repetition costs are used as a measure for inhibitory processes during task switching. On the other hand, congruency effects are supposed to reflect another mechanism, namely task shielding (Schuch & Grange, 2019). We do not postulate a causal relationship between these mechanisms. On practical reasons, the present design is not suited for such a conclusion, as all factors were manipulated concurrently within trials, so no clear causal direction can be derived. On theoretical reasons, we do not assume a direct causal relationship between inhibition and shielding. The reason for this is that both mechanisms manifest themselves on different trials, therefore operating at different timepoints and targeting different information. The target of inhibition underlying *n* − 2 repetition costs is the task that was relevant on trial *n* − 2, and this process is supposed to begin after the response of this trial, that is, during preparation for the task in trial *n* − 1 (e.g., Scheil & Kleinsorge, 2014; Schuch & Koch, 2003). The target of task shielding is the task set that is relevant in trial *n*, and this process is supposed to operate on trial *n* (e.g., Goschke & Dreisbach, 2008; Reisenauer & Dreisbach, 2014). This is further supported by the fact that only congruency in trial *n* interacted with the task sequence in the present study as well as in previous studies (Scheil & Kleinsorge, 2020). As a consequence, it seems unlikely that inhibition and task shielding directly affect each other. Rather, we propose that certain combinations of environmental demands put the cognitive system into different states that are more or less prone to interference and confusion. For example, in ABA sequences with incongruent stimuli, two factors play together: a still inhibited task set on the one hand and interfering information from the stimulus on the other hand. This interplay negatively affects task performance. However, as we cannot rule out a causal relationship based on the present study, we encourage future research to address this aspect in more detail.

An obvious consequence of using two response sets is that two consecutive switches result in a repetition of the response set from trial *n* − 2 to trial n. One could argue that this may have affected the results. Like it is the case for ABA sequences, the response set in trial *n* − 2 could have been inhibited, maybe even together with the task in this trial. If this inhibition persists to trial *n*, this would result in a delayed activation of the response set. However, we do not believe that this was the case in the present study. If activation of the response set was hampered in the double-switch condition, this should negatively affect task performance in this condition. However, this was not the case, neither with respect to the general RT level not regarding the size of *n* − 2 repetition costs. Therefore, we conclude that the double-switch condition of the response set does not confound the present data pattern. To vary the response set transition of three consecutive trials independently, a third response set would be necessary. However, this would further complicate the experiment, which is even in its current version quite complex. Therefore, we refrained from doing so.

We have to acknowledge that the present design is a rather complex one. As a consequence, the data pattern may be affected by other factors than inhibition and task shielding. We tried to rule out some of these factors, like episodic retrieval, response repetition effects, and congruency in previous trials. However, we cannot exclude that there are other factors, or a combination of them, that had an influence on the present results. Further research is needed to characterize the interplay of inhibition and shielding in task switching.

In conclusion, the present results support the notion of inhibition during task switching being affected by the degree of response set overlap. Overlapping response sets are more prone for interference and, as a consequence, have to be inhibited to a higher degree, causing higher *n* − 2 repetition costs. Furthermore, the present results offer additional support for the notion of an interplay of task inhibition and task shielding for the reduction of currently irrelevant information that depends on the demands of the task environment.

## Supplementary Information

Below is the link to the electronic supplementary material.

Supplementary file1 (DOCX 214 KB)

## Data Availability

Data will be made available on reasonable request.

## Footnotes

1 On theoretical reasons, one could also expect *n*-2 repetition costs to be affected by stimulus congruency in trial *n*-2, when activation of the to-be-inhibited task set takes place, or of congruency in trial *n*-1, when the same task set is assumed to be inhibited. However, neither congruency in trial *n*-2 nor in trial *n*-1 had any influence on the present data pattern. Most importantly, neither factor did interact with the task sequence. Therefore, the present data pattern seems to be specifically affected by stimulus congruency in trial *n*, not by congruency per se.

2 An extended version of this figure, including not only the data pattern but also example sequences of the different conditions, can be found in the online supplemental material.

## References

[CR1] Arbuthnott KD (2005). The influence of cue type on backward inhibition. Journal of Experimental Psychology: Learning, Memory, and Cognition.

[CR2] Dreisbach G (2012). Mechanisms of cognitive control: The functional role of task rules. Current Directions in Psychological Science.

[CR3] Dreisbach G, Wenke D (2011). The shielding function of task sets and its relaxation during task switching. Journal of Experimental Psychology: Learning, Memory, and Cognition.

[CR4] Faul F, Erdfelder E, Lang AG, Buchner A (2007). G* Power 3: A flexible statistical power analysis program for the social, behavioral, and biomedical sciences. Behavior Research Methods.

[CR5] Gade M, Koch I (2007). The influence of overlapping response sets on task inhibition. Memory & Cognition.

[CR6] Gade M, Koch I (2014). Cue type affects preparatory influences on task inhibition. Acta Psychologica.

[CR7] Goschke T, Dreisbach G (2008). Conflict-triggered goal shielding: Response conflicts attenuate background monitoring for prospective memory cues. Psychological Science.

[CR8] Grange JA, Juvina I, Houghton G (2013). On costs and benefits of n−2 repetitions in task switching: Towards a behavioural marker of cognitive inhibition. Psychological Research Psychologische Forschung.

[CR9] Grange JA, Kowalczyk AW, O'Loughlin R (2017). The effect of episodic retrieval on inhibition in task switching. Journal of Experimental Psychology: Human Perception and Performance.

[CR10] Heister, G., Schroeder-Heister, P., & Ehrenstein, W. H. (1990). Spatial coding and spatio-anatomical mapping: Evidence for a hierarchical model of spatial stimulus-response compatibility. In R. W. Proctor & T. G. Reeve (Eds.), *Stimulus-response compatibility* (pp. 117–143). North-Holland.

[CR11] Houghton G, Pritchard R, Grange JA (2009). The role of cue–target translation in backward inhibition of attentional set. Journal of Experimental Psychology. Learning, Memory, and Cognition.

[CR12] Jost K, Hennecke V, Koch I (2017). Task dominance determines backward inhibition in task switching. Frontiers in Psychology.

[CR13] Kiesel A, Steinhauser M, Wendt M, Falkenstein M, Jost K, Philipp AM, Koch I (2010). Control and interference in task switching—a review. Psychological Bulletin.

[CR14] Kleinsorge T (1999). Response repetition benefits and costs. Acta Psychologica.

[CR15] Koch I, Gade M, Schuch S, Philipp AM (2010). The role of inhibition in task switching: A review. Psychonomic Bulletin & Review.

[CR16] Koch I, Poljac E, Müller H, Kiesel A (2018). Cognitive structure, flexibility, and plasticity in human multitasking—An integrative review of dual-task and task-switching research. Psychological Bulletin.

[CR17] Kuhns D, Lien MC, Ruthruff E (2007). Proactive versus reactive task-set inhibition: Evidence from flanker compatibility effects. Psychonomic Bulletin & Review.

[CR18] Mayr U, Keele SW (2000). Changing internal constraints on action: The role of backward inhibition. Journal of Experimental Psychology: General.

[CR19] Nicoletti R, Umiltà C, Ladavas E (1984). Compatibility due to the coding of the relative position of the effectors. Acta Psychologica.

[CR20] Philipp AM, Koch I (2006). Task inhibition and task repetition in task switching. European Journal of Cognitive Psychology.

[CR21] Reisenauer R, Dreisbach G (2014). The shielding function of task rules in the context of task switching. Quarterly Journal of Experimental Psychology.

[CR22] Rogers RD, Monsell S (1995). Costs of a predictable switch between simple cognitive tasks. Journal of Experimental Psychology: General.

[CR23] Scheil J (2016). Effects of absolute and relative practice on n−2 repetition costs. Acta Psychologica.

[CR24] Scheil J, Kleinsorge T (2014). N− 2 repetition costs depend on preparation in trials n−1 and n−2. Journal of Experimental Psychology: Learning, Memory, and Cognition.

[CR25] Scheil J, Kleinsorge T (2019). Effects of global and local task repetition proportion on n−2 repetition costs. Quarterly Journal of Experimental Psychology.

[CR26] Scheil J, Kleinsorge T (2020). Further investigating effects of task repetition proportion on n-2 repetition costs: Task shielding as a potential modulating factor?. Quarterly Journal of Experimental Psychology.

[CR27] Scheil J, Kleinsorge T (2022). No-go trials in task switching: Effects on the task-set and task-space level. Psychological Research Psychologische Forschung.

[CR28] Schuch S, Grange JA (2019). Increased cognitive control after task conflict? Investigating the N−3 effect in task switching. Psychological Research Psychologische Forschung.

[CR29] Schuch S, Koch I (2003). The role of response selection for inhibition of task sets in task shifting. Journal of Experimental Psychology: Human Perception and Performance.

[CR30] Vandierendonck A (2018). Further tests of the utility of integrated speed-accuracy measures in task switching. Journal of Cognition.

[CR31] Vandierendonck A, Liefooghe B, Verbruggen F (2010). Task switching: Interplay of reconfiguration and interference control. Psychological Bulletin.

