# Supplementary material for: Effects of a dynamically changing response set overlap on n − 2 repetition costs
Source: Psychol Res. 2023 Mar 22;87(7):2275–82. doi: 10.1007/s00426-023-01816-w (PMC10457239; doi:10.1007/s00426-023-01816-w)
Supplement: Supplementary file 1 — Supplementary file1 (DOCX 214 KB) [file 426_2023_1816_MOESM1_ESM.docx]

Online Supplemental Material

**Fig. 1** Schematic illustration of the procedure of a single trial. After presentation of the fixation mark, the example task cue refers to the size task, while the color indicated the relevant response set. After 600ms, the imperative stimulus is presented (in this case, a large one) and stays on the screen together with the task cue until a response is given.

**Fig. 2** Extended depiction of data pattern and trial sequences of Experiment I. Data of the four-way interaction are shown in the middle of each section. Examples of trial sequences are shown on the left and right, corresponding to the respective conditions in the graph. A, B, and C denote the relevant task in trials *n* – 2, *n* – 1, and *n*. Green color indicates congruent stimuli, red color indicates incongruent stimuli. Each trial sequence begins with the left response set by way of example.


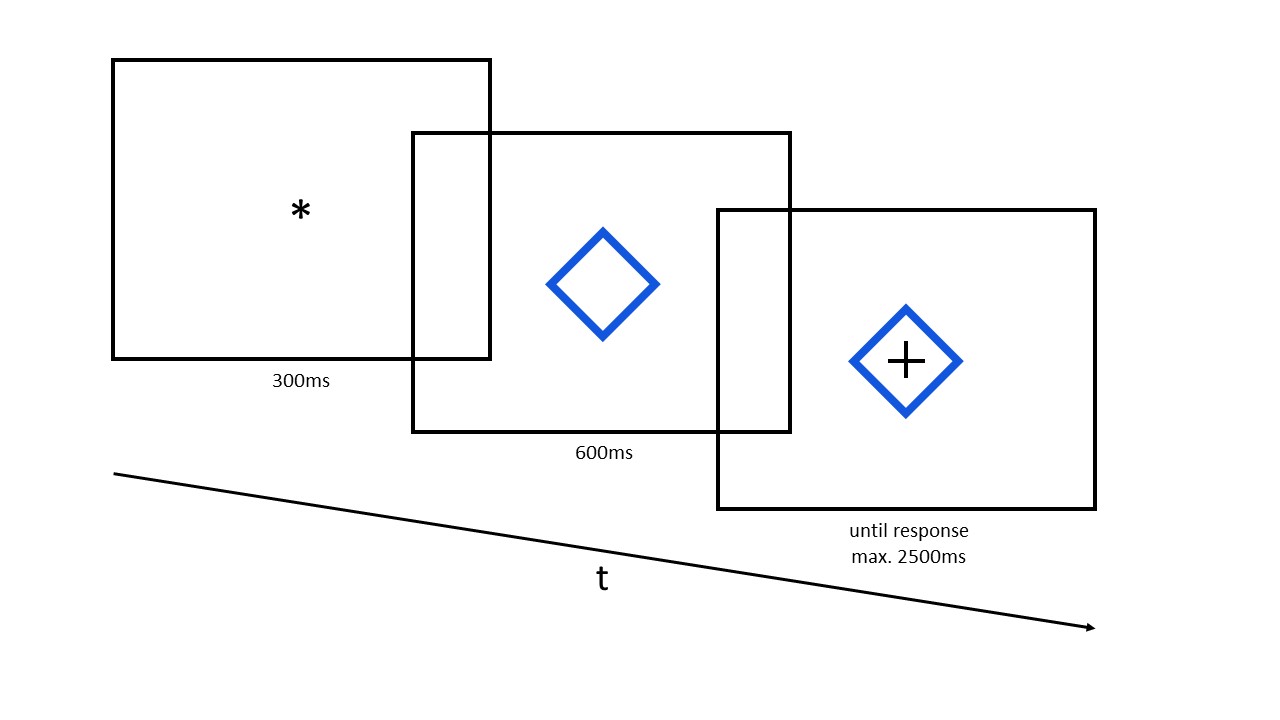


Figure 1


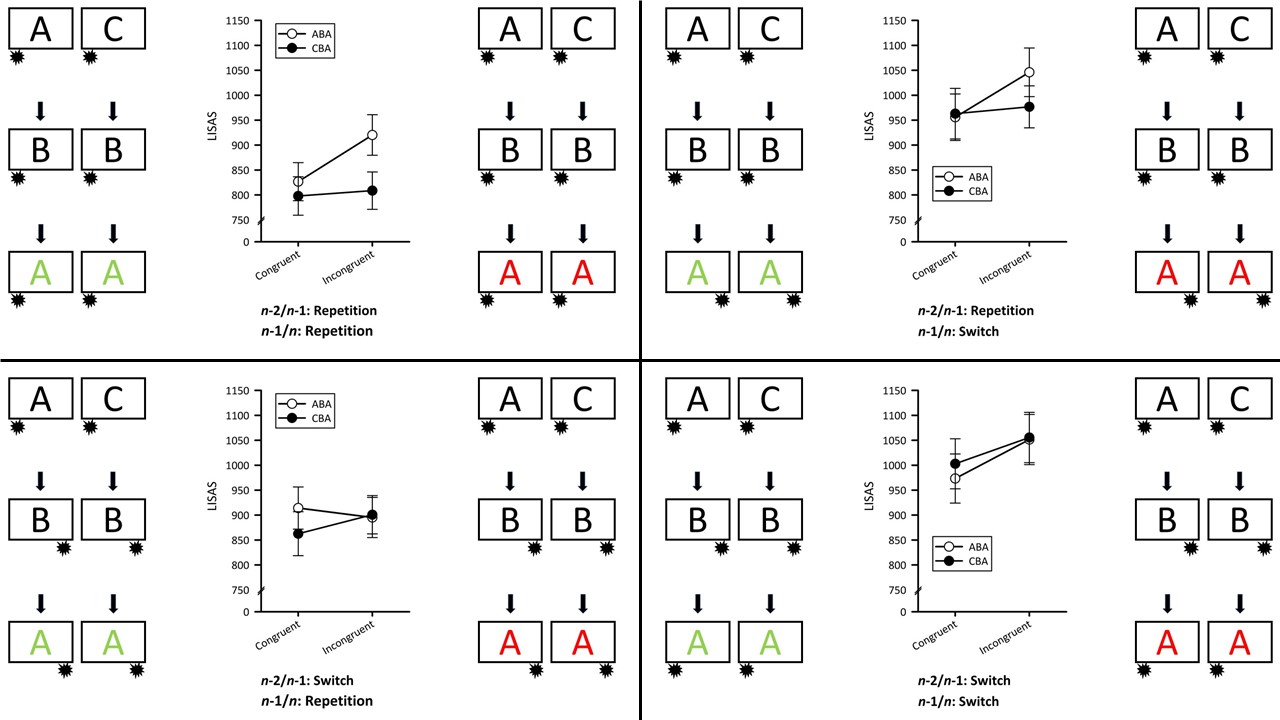


Figure 2
